# Preterm‐birth alters the development of nodal clustering and neural connection pattern in brain structural network at term‐equivalent age

**DOI:** 10.1002/hbm.26442

**Published:** 2023-08-04

**Authors:** Weihao Zheng, Xiaomin Wang, Tingting Liu, Bin Hu, Dan Wu

**Affiliations:** ^1^ Gansu Provincial Key Laboratory of Wearable Computing, School of Information Science and Engineering Lanzhou University Lanzhou China; ^2^ Key Laboratory for Biomedical Engineering of Ministry of Education, College of Biomedical Engineering & Instrument Science Zhejiang University Hangzhou China; ^3^ School of Medical Technology Beijing Institute of Technology Beijing China; ^4^ CAS Center for Excellence in Brain Science and Intelligence Technology, Shanghai Institutes for Biological Sciences Chinese Academy of Sciences Shanghai China; ^5^ Joint Research Center for Cognitive Neurosensor Technology of Lanzhou University & Institute of Semiconductors Chinese Academy of Sciences Lanzhou China

**Keywords:** classification, graph theory, preterm, structural network, term‐equivalent age

## Abstract

Preterm‐born neonates are prone to impaired neurodevelopment that may be associated with disrupted whole‐brain structural connectivity. The present study aimed to investigate the longitudinal developmental pattern of the structural network from preterm birth to term‐equivalent age (TEA), and identify how prematurity influences the network topological organization and properties of local brain regions. Multi‐shell diffusion‐weighted MRI of 28 preterm‐born scanned a short time after birth (PB‐AB) and at TEA (PB‐TEA), and 28 matched term‐born (TB) neonates in the Developing Human Connectome Project (dHCP) were used to construct structural networks through constrained spherical deconvolution tractography. Structural network development from preterm birth to TEA showed reduced shortest path length, clustering coefficient, and modularity, and more “connector” hubs linking disparate communities. Furthermore, compared with TB newborns, premature birth significantly altered the nodal properties (i.e., clustering coefficient, within‐module degree, and participation coefficient) in the limbic/paralimbic, default‐mode, and subcortical systems but not global topology at TEA, and we were able to distinguish the PB from TB neonates at TEA based on the nodal properties with 96.43% accuracy. Our findings demonstrated a topological reorganization of the structural network occurs during the perinatal period that may prioritize the optimization of global network organization to form a more efficient architecture; and local topology was more vulnerable to premature birth‐related factors than global organization of the structural network, which may underlie the impaired cognition and behavior in PB infants.

## Graphical abstract

Our findings demonstrate a topological reorganization of the structural network occurs during the perinatal period that may prioritize the optimization of global network organization to form a more efficient architecture; and local topology is more vulnerable to premature birth related factors than global organization of the structural network.

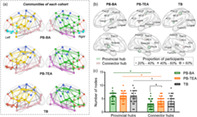

## 1 INTRODUCTION

Human brain experiences rapid and complex growth over the perinatal period, characterized by dynamic macro‐ and micro‐structural maturation, such as increasing axonal density and myelination, volume expansion, and formation of large‐scale brain connectome (Ball et al., 2013; Fenchel et al., 2020; Jaimes et al., 2020; Ouyang et al., 2019; Wu et al., 2023; Zhao et al., 2019). These changes set the fundamental anatomical architecture in the brain that is essential for supporting high‐order cognitive functions, for example, fiber tracts build bridges between isolated brain regions that are fundamental for efficient cognitive processing. Preterm‐born is known to disrupt the typical brain development, especially in the white matter (WM), leading consequently to later motor, cognitive, and behavioral deficits and may raise the risk for mental disorders (Fischi‐Gomez et al., 2016; Hallin et al., 2010; Joseph et al., 2016; Montagna & Nosarti, 2016; Srinivas Jois, 2018; Young et al., 2018). Characterizing alterations of WM connections during this period is of great importance for understanding normative brain development and elucidating the association between cerebral dysmaturation and premature encephalopathy.

Major fiber tracts have been delineated at the beginning of the second trimester via in vivo and ex vivo studies (Huang et al., 2006; Huang et al., 2009; Song et al., 2015; Takahashi et al., 2012; Vasung et al., 2010; Wilson et al., 2021; Zheng et al., 2023). For example, commissural and projection fibers appear first (Huang et al., 2009; Takahashi et al., 2012), and then axons within association tracts (e.g., inferior longitudinal fasciculus) form at around 15–17 gestation weeks (Huang et al., 2006; Kostovic & Judas, 2006). Also during this critical period, preterm‐birth and other perinatal insults may lead to WM injury and hypomyelination (Ortinau & Neil, 2015; Wang et al., 2019), for example, impaired myelination in the posterior limb of internal capsule is recognized as a sign of developmental delay in very preterm‐born infants (McArdle et al., 1987). Recent studies indicated that preterm birth significantly alters the typical developmental trend of the brain, for example, preterm‐born neonates showed increased cortical tissue water content and reduced neurite density compared with full‐term infants, and the deviations from the typical microstructural development were highly heterogeneous between individual preterm‐born neonates (Dimitrova et al., 2020; Dimitrova et al., 2021; Pannek et al., 2018).

Other studies have begun to characterize WM development of premature neonates from a network organization perspective (Ball et al., 2014; Batalle et al., 2017; Brown et al., 2014; Jang et al., 2023; Sa de Almeida et al., 2021; van den Heuvel et al., 2015; Young et al., 2018; Zhao et al., 2019), using graph theory to depict the topological integration and segregation of a network via a series of quantitative measures (Bullmore & Sporns, 2009; Rubinov & Sporns, 2010). For example, prematurity might disrupt WM connectivity and alter the topology of structural networks, manifested as increased modularity and clustering coefficient, decreased global efficiency, and weakened connectivity among rich‐club nodes, at term‐equivalent age (TEA) in comparison to full‐term borns (Ball et al., 2014; Batalle et al., 2017; Pandit et al., 2014; Sa de Almeida et al., 2021). Some other studies demonstrated opposite results, for example, increased local clustering of the structural network during perinatal brain development (Ball et al., 2014; Brown et al., 2014; van den Heuvel et al., 2015; Zhao et al., 2019), or comparable network organization between preterm and term‐born neonates (Brown et al., 2014). Nonetheless, given the inconsistent findings, how early exposure to the extrauterine environment alters the typical developmental trajectory of WM topology in premature brains remains an open question.

The present study aimed to investigate the longitudinal topological changes of the structural network from preterm birth to TEA and the impact of preterm birth on the typical network architecture at TEA. Multi‐shell high angular resolution diffusion imaging (HARDI) data of 28 pairs of well‐matched preterm‐born neonates and term newborns from the Developing Human Connectome Project (dHCP) were included. We calculated graph‐theoretic measures at global, regional, and modular levels to comprehensively characterize the longitudinal developmental pattern from preterm birth to TEA and the prematurity‐related alterations in structural network organization by comparing the preterm‐ and term‐born pairs at TEA. A classification model was trained based on the network measures to examine the separability of preterm‐ and term‐born infants at TEA. Correlations between network topology and WM microstructures were computed to elucidate the structural basis of network alteration.

## 2 MATERIALS AND METHODS

### 2.1 Participants

Neonates included in this study were recruited as a part of the dHCP (REC: 14/LO/1169, http://www.developingconnectome.org/second-data-release/). We initially included 40 preterm‐born (PB) neonates who were scanned twice several days after birth (PB‐AB, 29–36 weeks postmenstrual age [PMA]) and term‐equivalent age (PB‐TEA, 38–44 weeks PMA), respectively. Twelve PB neonates were excluded due to the evident motion artifacts and intracranial abnormality (radiology score >3, an evaluation provided by the dHCP). For each PB‐TEA infant, we selected a healthy term‐born (TB) neonate who had matched gender, PMA at scan, and head circumference at scan with the PB‐TEA. Finally, a total of 28 PB neonates, who had scans at both birth and TEA and 28 matched TB neonates were included. Basic information regarding the demographics of the participants is given in Table 1.

**TABLE 1:** Demographic and basic clinical information of infants included in this study.

Clinical characteristics	PB‐AB	PB‐TEA	TB
Gestational age at birth (weeks)	25.57–36.00 (32.43 ± 2.86)	–	37.57–41.86 (39.98 ± 1.21)
Postmenstrual age at scan (weeks)	29.86–36.86 (34.57 ± 1.60)	38.43–44.86 (41.29 ± 1.47)	38.43–44.71 (41.29 ± 1.45)
Sex: male/female	17/11	–	17/11
Birth weight (kg)	1.80 ± 0.69	–	3.44 ± 0.56
Head circumference (cm)	30.09 ± 2.55	35.29 ± 1.66	35.38 ± 1.24
Singleton/twins	18/10	–	28/0
Radiology score (No. of 1/2/3)	15/7/6	9/8/11	16/12/0

Abbreviations: PB‐AB, preterm‐born neonates scanned after birth; PB‐TEA, preterm‐born neonates scanned at term‐equivalent age; TB, term‐born neonate.

### 2.2 MRI acquisition and preprocessing

Images were acquired on a 3‐Tesla Philips Achieva scanner equipped with a 32‐channel neonatal head coil and a baby transportation system (Hughes et al., 2017). Multi‐shell diffusion‐weighted MRI (dMRI) was acquired with the following parameters: sampling *b*‐values of *b* = 0, 400, 1000, and 2600 s/mm^2^, with spherically uniformly distributed 20, 64, 88, and 128 directions, respectively; repetition time (TR)/echo time (TE) = 3800/90 ms; multi‐band acceleration factor = 4; in‐plane resolution = 1.5 × 1.5 mm, and 3 mm slice thickness with 1.5 mm overlap (Hutter et al., 2018). The total examination time for diffusion MRI is 19 min 20 s for a baby (Edwards et al., 2022).

The downloaded images were preprocessed through the dHCP diffusion image processing pipeline (Bastiani et al., 2019) and were controlled for quality via the EDDY QC tool (Bastiani et al., 2019). The preprocessing steps included intra‐subject volume registration (Jenkinson et al., 2002), distortion correction, and eddy current correction using FSL tools (Andersson et al., 2003; Andersson et al., 2017; Andersson & Sotiropoulos, 2016), and super‐resolution reconstruction along the slice‐selection direction (Kuklisova‐Murgasova et al., 2012) to achieve 1.5 mm isotropic resolution. A summary of the image quality metrics calculated by the EDDY QC tools is shown in Table S1.

### 2.3 Network construction

The nodes of networks were defined by the neonatal Automated Anatomical Labeling (AAL) atlas proposed by (Shi et al., 2011), which contains 90 cortical and subcortical regions. A 12‐parameter affine registration was applied to transform the T2 template image in the AAL atlas space to the b0 images in the native dMRI space, followed by a non‐linear registration, to achieve brain parcellation in the native space. The connections between brain regions were obtained using a probabilistic fiber tracking algorithm based on the second‐order integration over fiber orientation distributions (iFOD2) computed by the MSMT‐CSD algorithm in MRtrix3 software (Jeurissen et al., 2014; Tournier et al., 2010). This approach addresses the challenge of resolving crossing fibers within a voxel and is more accurate in tractography mapping than the diffusion tensor model. Specifically, 10 million streamlines were generated across the whole brain with the following tracking parameters: step size = 0.5 mm, maximum angle = 22.5°, min/max length = 10/250 mm, and cutoff = 0.05 (Liu et al., 2021). The streamlines were filtered to 1 million using spherical‐deconvolution informed filtering of tractograms (SIFT) to reduce tractography bias. A structural connectivity matrix (90 × 90) was generated by counting the number of filtered streamlines between brain regions. The connectivity matrix was normalized through a fractional scaling strategy (Cheng et al., 2012; Donahue et al., 2016) using the average number of streamlines between all pairs of nodes. The weakest 5% of the structural connections, which were considered spurious streamlines, were discarded from the analysis.

The length of each edge was calculated by averaging all traced fibers connecting two nodes associated with this edge. This step was performed after registering individual brains to a population brain template. We categorized the edges into short‐ and long‐range connections through an edge length threshold (Zhao et al., 2019). Networks based on short‐ and long‐range connections were constructed, respectively, for each subject to characterize the developmental pattern of short‐ and long‐range paths.

### 2.4 Network analysis

Network metrics were calculated based on the normalized weighted networks using the Brain Connectivity Toolbox (Rubinov & Sporns, 2010). Four common global properties that reflect the local and global organization as well as the architecture of the network, including the shortest path length (a measure of graph integration), clustering coefficient (a measure of graph segregation), small‐worldness (evaluates the network organization compare to a matched random graph), and modularity (measures the decomposability of a graph into several sparsely interconnected communities), were extracted from weighted networks. Regional clustering coefficient that measures nodal efficiency (Latora & Marchiori, 2003), as well as within‐module degree and participation coefficient that reflect intra/inter‐modular connectivity of a node, were also computed for characterizing nodal topology.

#### 2.4.1 Global network metrics

For a weighted network, a path length is the total sum of individual link lengths, and the link lengths are inversely related to link weights (Rubinov & Sporns, 2010). The shortest path length between two brain regions measures the efficiency of information transfer between the two regions. The shortest weighted path length between node *i* and *j* is formulated as:

$$L_{\mathrm{ij}}^{w}=\sum_{a_{\mathrm{uv}}\in g_{i↔j}^{w}}f\left(w_{\mathrm{uv}}\right)$$

where *f* is a map from weight to length, and $g_{i↔j}^{w}$ is the shortest weighted path between *i* and *j*.

The clustering coefficient is defined as:

$$C^{w}=\frac{1}{n}\sum_{i\in N}\frac{2}{k_{i}\left(k_{i}-1\right)}\sum_{j,k}{\left(w_{\mathrm{ij}}^{\prime }w_{\mathrm{jk}}^{\prime }w_{\mathrm{ki}}^{\prime }\right)}^{\frac{1}{3}}$$

where *k*_*i*_ and *t*_*i*_ represent the degree of node *i* and the number of triangles attached to node *i*, respectively; the weights of neighbor edges were scaled by the largest weight in the network, $w_{\mathrm{ij}}^{\prime }=w_{\mathrm{ij}}/\max\left(w_{\mathrm{ij}}\right)$. Therefore, the weighted clustering coefficient is the unweighted clustering coefficient renormalized by the average intensity of triangles at the node. The average clustering coefficient is the average of this value across nodes.

In general, a small‐world network is characterized by higher clustering and comparable efficiency relative to a matched random graph (Watts & Strogatz, 1998). The weighted network small‐worldness is defined as:

$$S^{w}=\frac{C^{w}/C_{\mathrm{rand}}^{w}}{L^{w}/L_{\mathrm{rand}}^{w}}$$

where $C_{\mathrm{rand}}^{w}$ and $L_{\mathrm{rand}}^{w}$ are the weighted clustering coeffificients and the shortest weighted path lengths of a random network, respectively.

These network properties have shown evident lateralization in neonatal brains (Ratnarajah et al., 2013), suggesting topological asymmetry might be a valuable feature to characterize brain development. To investigate the developmental pattern of topological lateralization of the structural network, we performed connectivity analysis in the left and right hemispheres separately (45 × 45 nodes for each matrix) based on the whole brain tractography (Ratnarajah et al., 2013), and compared the network topological properties (i.e., clustering coefficient, the shortest path length, small‐worldness, and modularity) between left and right hemispheres.

#### 2.4.2 Module detection

The module detection algorithm was performed on the normalized weighted network using a similar pipeline as described in (Cohen & D'Esposito, 2016; Zheng et al., 2019; Zheng et al., 2021). Briefly, we utilized the Louvain community detection algorithm with default parameter (gamma = 1) to estimate the optimal modular organization in the network (Blondel et al., 2008). A consensus matrix (*D*) was calculated by repeating the algorithm 150 times, where *D*_*ij*_ indicated the probability that node *i* and node *j* were assigned to the same community. We set the agreement value to 0 if the probability was lower than 50%. We then ran the Louvain algorithm on the thresholded consensus matrix (*D*) to estimate the final modular partition. This module detection pipeline was suggested to be more robust than other commonly used approaches (Lancichinetti & Fortunato, 2012). Modularity measures the extent to which a network can be decomposed into non‐overlapping communities with maximized intra‐module edges and minimized inter‐community edges, formulated as:

$$Q^{w}=\frac{1}{l^{w}}\sum_{i,j\in N}\left[w_{i,j}-\frac{k_{i}^{w}k_{j}^{w}}{l^{w}}\right]\delta_{m_{i},m_{j}}$$

where *l* ^*w*^ is the sum of all weights in the network, *w*_*ij*_ is the connection weight between node *i* and *j*, $k_{i}^{w}$ is the weighted degree of node *i*, and *m*_*i*_ is the module to which node *i* belongs ($\delta_{m_{i},m_{j}}=1$ if *m*_*i*_ = *m*_*j*_, and 0 otherwise).

#### 2.4.3 Hub analysis

Within‐module degree and participation coefficient were calculated as measures related to the role each hub node plays in the network (Guimerà & Nunes Amaral, 2005). The within‐module degree of node *i* is defined as $\frac{k_{i}^{m}-{\bar{k}}^{m}}{\sigma^{k^{m}}}$, where $k_{i}^{m}$ is the weighted degree of node *i* within its own module (*m*), ${\bar{k}}^{m}$ and $\sigma^{k^{m}}$ are the average and standard deviation of the within‐module *m* degree distribution. The participation coefficient of node *i* is defined as $1-\sum_{m=1}^{M}{\left(\frac{k_{i}^{m}}{k_{i}}\right)}^{2}$, where *M* is the number of modules and *k*_*i*_ is the total weighted degree of node *i*. These two measures were utilized to categorize hub nodes into “provincial” hubs (hubs that predominantly connect to other nodes in the same community) and “connector” hubs (hubs that link different communities together). Here, we defined provincial hubs as those with *z*‐scored *z* (within‐module degree) ≥ 1 and participation coefficient <0.3; and connector hubs as those with *z* (within‐module degree) < 1 and participation coefficient ≥0.3 (Guimerà & Nunes Amaral, 2005). For each group, nodes with the ratio of subjects who owned these nodes as provincial / connector hubs over 20% were visualized to represent the spatial distribution of hub regions. Due to the lack of an optimized threshold for the infant, we chose a widely used adult‐based threshold (participation coefficient = 0.3) for hub categorization, which might not fit infant brains and need to be further tested in larger samples.

### 2.5 dMRI‐based microstructural metrics

Fractional anisotropy (FA) that describes the degree of anisotropy was computed through MRtrix3 software. The FA was averaged within the whole brain and each fiber connection.

### 2.6 Influences of network sparsity and motion artifacts

To characterize the robustness of network analyses, we calculated global network metrics on binary networks with varying network sparsity (retaining the strongest 10%–25% of links in 5% increments) and set suprathreshold links (edges) to 1 and the rest to 0. Group comparisons were made at each sparsity. We also examined the influence of head motion on network analysis by controlling the absolute head motion of individuals during statistical analysis.

### 2.7 Classification analysis

We performed a classification analysis to investigate whether PB neonates at TEA can be identified from TBs through the network topological properties (i.e., clustering coefficient, within‐module degree, and participation coefficient of brain regions). Topological properties of all brain regions were cascaded to a vector (dimension = 1 × 270). We applied principal component analysis (PCA) for feature reduction. We determined the number of principle components (~30) that captured over 90% variance of the data for feature reduction. A linear support vector machine (SVM) with default parameters was used for classification. Classification performance was evaluated via leave‐one‐out cross validation due to the small sample size. In addition, five‐fold and three‐fold cross‐validation strategies were performed for comparison, which were repeated for 10 times. Note that the PCA was performed on the training set during the cross‐validation process. For data visualization, we performed the PCA on the data matrix of clustering coefficient, within‐module degree, and participation coefficient, respectively; and projected samples to a low dimensional space based on the first principal component of each feature (i.e., clustering coefficient, within‐module degree, and participation coefficient).

### 2.8 Statistical analysis

#### 2.8.1 Group comparison analysis

We performed paired t‐test and analysis of covariance (ANCOVA) to examine the group differences between PB‐AB and PB‐TEA and between PB‐TEA and TB, respectively. In the ANCOVA model, a multiple regression technique was used, with age at scan, head circumference at scan, and the number of gestation embryos (considering the twin effect [Liu, Zhang, et al., 2021]) as covariates. Paired t‐test was also used to compare the network difference between the two hemispheres. The false discovery rate (FDR) method at the level of *q* = 0.05 was utilized for correcting multiple comparisons.

#### 2.8.2 Correlation analysis

A cross‐subject partial correlation analysis, with the aforementioned covariates, between network metrics (i.e., clustering coefficient and shortest path length) and whole‐brain averaged FA was performed in two cohorts, including the longitudinal cohort from preterm‐birth to TEA (PB‐AB + PB‐TEA) and paired cross‐sectional cohort at full‐term age (PB‐TEA + TB).

#### 2.8.3 Randomization test

To examine whether the classification performance we achieved was significantly higher than the chance level, we performed a randomization test to generate a confidence interval (CI) of chance‐level accuracy via repeating the cross‐validation procedure 1000 times with randomly shuffled labels (Zheng et al., 2021). The real accuracy exceeded 95% CI indicating the classification performance significantly outperformed random guessing.

## 3 RESULTS

### 3.1 Group differences in global network topology

**FIGURE 1:**
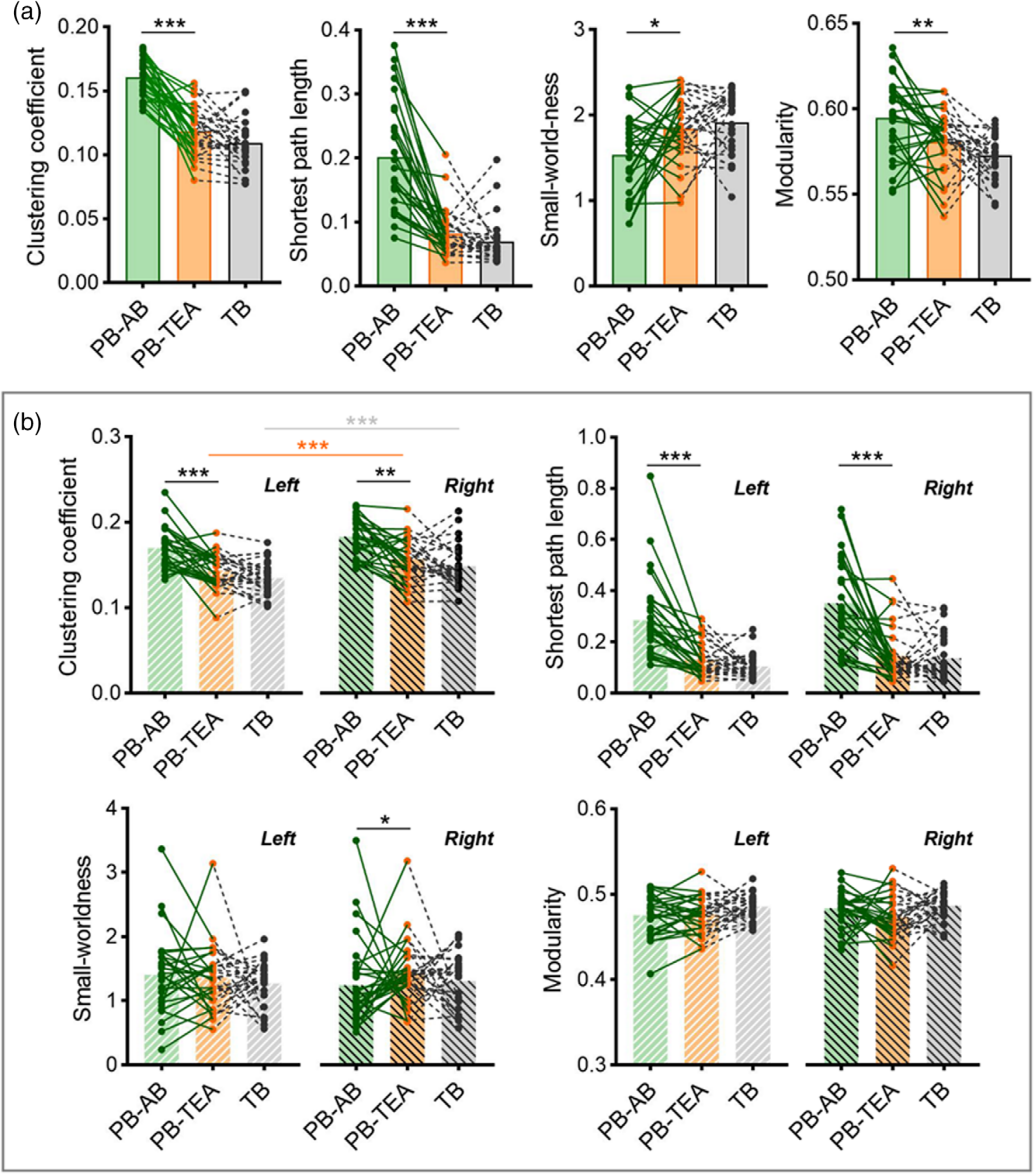
Comparison of global network topology between groups. (a) Between‐group differences in clustering coefficient, the shortest path length, small‐worldness, and modularity. Compared with PB‐AB, PB‐TEA showed significant increases of global efficiency and small‐worldness, and significant decreases of clustering coefficient and modularity. No significant difference was found in these metrics between PB‐TEA and TB cohorts. (b) Comparison of clustering coefficient, global efficiency, small‐worldness, and modularity between left and right hemispheres. Clustering coefficient of PB‐TEA and TB showed significant right‐ward lateralization, whereas no significant inter‐hemispherical difference was found in other network metrics. The green line depicts the longitudinal changes of a subject from preterm‐birth to TEA, while the black dash line indicates the paired relation between a PB‐TEA infant and a matched TB neonate. *p < 0.05, **p < 0.01, ***p < 0.001 by paired *t*‐test for longitudinal and inter‐hemispherical analysis and ANCOVA for PB‐TEA vs. TB.

All the structured networks were fully connected, with the average connective density of 0.24 ± 0.03 for PB‐AB, 0.32 ± 0.03 for PB‐TEA, and 0.33 ± 0.02 for TB, respectively. Significant decreases of clustering coefficient and the shortest path length were observed (paired t‐test, *p* < .001), accompanied by increased small‐worldness and reduced modularity (paired t‐test, *p* < .05) during the network development from preterm‐birth to TEA in PB neonates (Figure 1a). However, no significant difference was found in these network metrics between the PB‐TEA and TB neonates (ANCOVA, *p* > .05). Similary alteration pattern of these network metrics were also observed in binary networks at different sparsity (Figure S1), and head motion did not significantly affect the statistic results, though the absolute motion of PB‐TEA was higher than PB‐AB (*p* < .05, Figure S2).

We further investigated whether the network topology exhibited an asymmetric pattern between hemispheres. As shown in Figure 1b, significant right‐ward lateralization of clustering coefficient was found in PB‐TEA and TB cohorts (paired *t*‐test, *p* < .001), whereas, this asymmetric pattern was not observed in PB neonates at birth. The global efficiency, small‐worldness, and modularity did not show significant asymmetry in the three groups.

### 3.2 Spatiotemporal changes of nodal properties across brain regions

**FIGURE 2:**
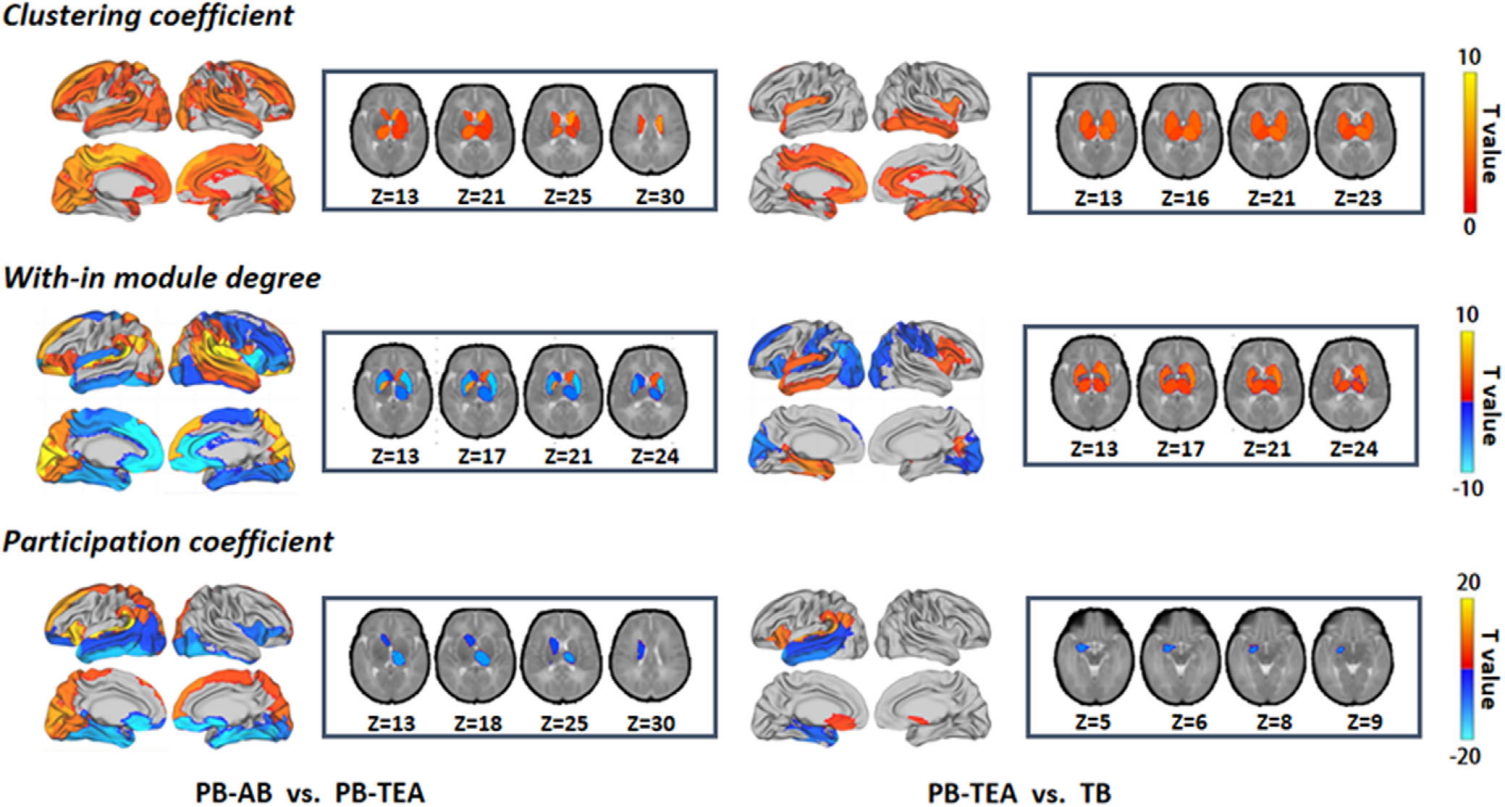
Group differences in clustering coefficient, within‐module degree, and participation coefficient between preterm‐born neonates scanned at birth (PB‐AB) and term‐equivalent age (TEA), and between PB‐TEA and term‐born (TB) neonates. Red or blue indicates the higher or lower values in the former compared with those in the latter. We conducted paired *t*‐test for longitudinal analysis and ANCOVA for PB‐TEA vs. TB. Multiple comparisons were corrected by the FDR approach and values with *q* < 0.05 were displayed.

More significant differences were found in nodal properties rather than the global topology of the network. Figure 2 illustrates the comparisons of clustering coefficient, within‐module degree, and participation coefficient among the three groups. A significant drop in nodal clustering was observed across the whole brain during the development of PB brains from birth to term (paired t‐test, *q* < 0.05, FDR corrected). Moreover, we found clustering coefficient was lower in subcortical nuclei and insular, cingulate, and inferior temporal cortices in TB neonates compared with the PB‐TEA cohort (ANCOVA, *q* < 0.05, FDR corrected). The within‐module degree and participation coefficient both showed reductions from the first to the second scan in the PB group, primarily in the left dorsolateral prefrontal cortex, left insular cortex, right dorsomedial prefrontal cortex, precuneus, and medial occipital cortex, and enhancements in the right insular cortex, medial inferior frontal cortex, medial temporal cortex, and subcortical nuclei (paired t‐test, *q* < 0.05, FDR corrected). Besides, increased within‐module degree was observed in medial frontal and parietal cortices of the PB infants at TEA compared with that at birth (paired *t*‐test, *q* < 0.05, FDR corrected). Similar alteration pattern of participation coefficient in left insular and temporal cortices when comparing the PB‐TEA to TB neonates (ANCOVA, *q* < 0.05, FDR corrected). The comparison results of clustering coefficient and participation coefficient indicated a gradual development of the brain structural network from PB‐AB to PB‐TEA to TB.

### 3.3 Changes in modular organization and hub distribution

**FIGURE 3:**
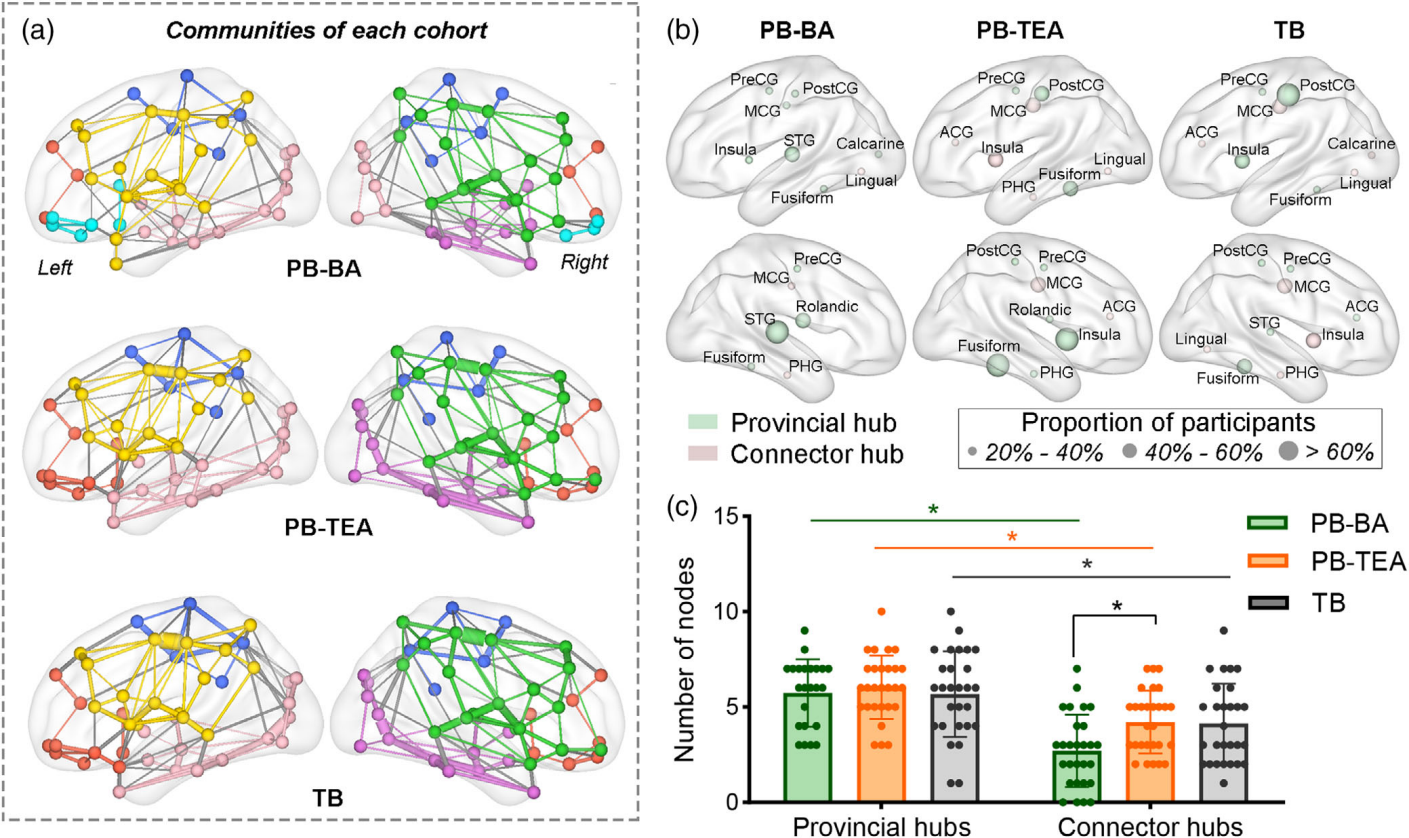
Modular and hub organization of each group. (a) Visualization of the modules detected in each group. Colors indicate different modules. (b) Spatial distribution of hub regions. Nodes in light green and pink indicate provincial and connector hubs, respectively. Size of a node represents the proportion of participants who owned this node as provincial or connector hub in each group. (c) Comparison of the number of connector and provincial hubs. We conducted paired *t*‐test for longitudinal analysis and ANCOVA for PB‐TEA vs. TB. * FDR *q* < 0.05.

The consistent modular organization was found across the three groups (Figure 3a), which can be spatially categorized into five densely intra‐connected communities, including prefrontal (in red), parietal (in blue), left and right dorsolateral (in yellow and green, respectively), and left and right ventral (in pink and violet, respectively) subsystems. Besides, a unique module located in the orbital parts of the inferior frontal gyrus was identified only in the PB‐AB cohort (in cyan), which was merged with the prefrontal community later at TEA. Figure 3b illustrated the spatial distribution of provincial and connector hubs in the three groups. Over 60% of PB‐AB neonates owned right STG as the provincial hub and other primary provincial hubs were left STG and right rolandic operculum (ratio of participants >50%), fusiform (ratio of participants >34%), precentral and postcentral gyri (PreCG and PostCG, with the ratio of 28% and 39%, respectively), left middle cingulate gyrus (MCG, 28% individuals), and left insula (25% individuals); whereas, connector hubs were primarily located in left lingual gyrus, right MCG and parahippocampus (PHG) that showed less generalizability across individuals (<32% individuals). Compared with the PB‐AB, both PB‐TEA and TB groups showed an increased number of connector hubs (*p* < .05, FDR corrected, Figure 3c), and the location of connectors of the two groups was highly consistent, for example, bilateral MCG (>50% individuals), insula, anterior cingulate gyrus (ACG, >32% individuals), and lingual gyrus (>32% individuals) became connector hubs in PB‐TEA and TB groups. Although no significant differences in provincial hubs were found among the three cohorts, STG was no longer the main provincial hub in PB‐TEA and TB cohorts, and PostCG, fusiform, and insula became more important within communities. These findings might suggest a modular reorganization occurs during the third trimester to optimize the modular structure and global coordination of information flow over the whole network, for example, densely intra‐connected modules with more connectors to enable efficient inter‐module communication.

### 3.4 Development of short‐ and long‐range fibers and the relationship between fiber integrity and network metrics

**FIGURE 4:**
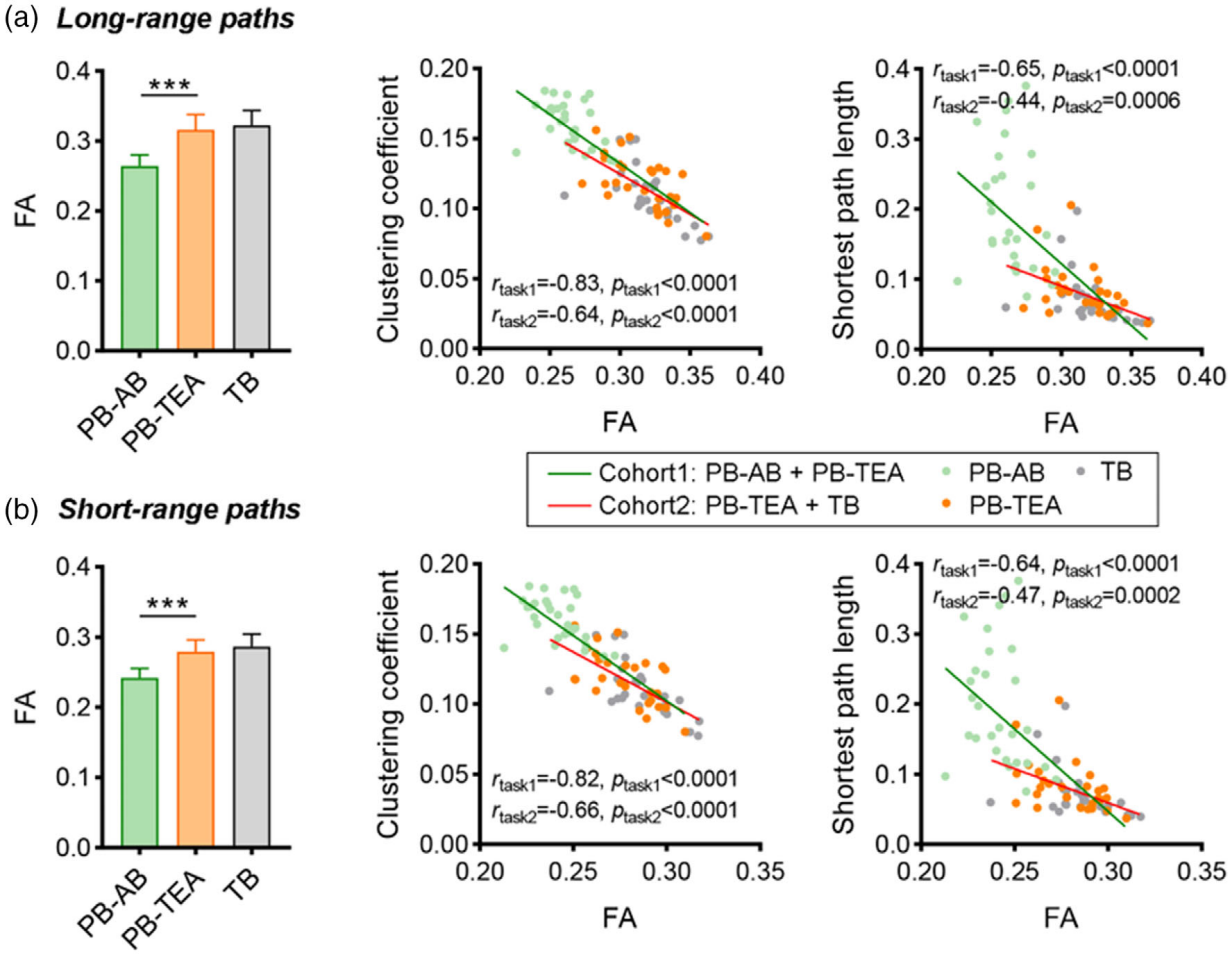
Comparison of FA in (a) short‐range and (b) long‐range connections, and its association with network metrics during brain development. Network metrics (i.e., clustering coefficient and shortest path length) in (a) and (b) were extracted from networks built upon short‐ and long‐range connections, respectively. We conducted paired *t*‐test for longitudinal analysis and ANCOVA for PB‐TEA vs. TB. The cross‐subject partial correlations between FA and these network metrics were evaluated in a longitudinal cohort (PB‐AB + PB‐TEA) and a paired cross‐sectional cohort at full‐term age (PB‐TEA + TB). ***p < .001.

As shown in Figure 4, mean FA of both short‐ and long‐range connections significantly increased in PB‐TEA infants compared with PB‐AB neonates (Figure 4, paired *t*‐test, *p* < .001); whereas no significant difference was found in FA of these connections between the PB‐TEA infants and TB newborns (ANCOVA, *p* > .05). For networks constructed based on only long‐range connections, we found that the mean FA of the entire brain negatively correlated with both clustering coefficient (*r* ≤ −0.64, *p* < .0001) and the shortest path length (*r* ≤ −0.44, *p* ≤ .0006) during the preterm‐birth to TEA development (PB‐AB + PB‐TEA) and at term age (PB‐TEA + TB). Similarly, metrics of short‐range connectivity networks showed negative correlations with FA (*r* ≤ −0.66, *p* < .0001 for clustering coefficient; and *r* ≤ −0.47, *p* ≤ .0002 for the shortest path length) in both of the two cohorts (i.e., PB‐AB + PB‐TEA and PB‐TEA + TB).

### 3.5 Classification performance of PB‐TEA versus TB using nodal topological properties

**FIGURE 5:**
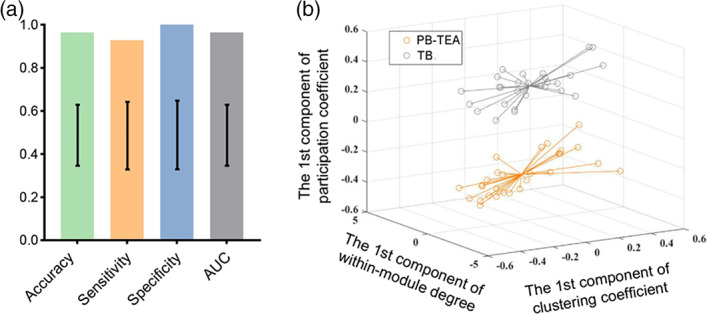
Classification performance of PB‐TEA and TB neonates using nodal topological features. (a) Classification performance derived from the leave‐one‐out cross‐validation. Classification accuracy of 96.43% was achieved in separating PB‐TEA and TB neonates, which significantly surpassed random guessing. The error bars represent the 5% and 95% CI of chance accuracy distribution estimated by randomization analysis. (b) Projection of subjects in the first principal component space of each feature. Principal component analysis was performed on the data matrix of clustering coefficient, within‐module degree, and participation coefficient, respectively. Samples were projected to low dimensional space by using the first principal component of each feature (clustering coefficient, within‐module degree, and participation coefficient). A clear boundary between the two groups can be identified on the axis of participation coefficient projection.

Using the nodal topological properties (i.e., clustering coefficient, within‐module degree, and participation coefficient), we achieved 96.43% accuracy in identifying PB‐TEA from TB neonates from the leave‐one‐out cross‐validation, with sensitivity/specificity = 92.86%/100% and area under the receiver operating characteristic curve (AUC) = 0.9643 (Figure 5a). The classification performance maintained stable across multiple cross‐validation strategies (i.e., leave‐one‐out, five‐fold, and three‐fold), with average accuracies of 97.32% (AUC = 0.9749) and 97.29% (AUC = 0.9709) for three‐ and five‐fold cross‐validation, respectively. The real accuracy exceeded 95% CI of the accuracy at the chance level, suggesting the statistical significance of the classification performance. The distribution of subjects projected in the feature space through the first principal component of each feature showed a clear boundary between PB‐TEA neonates and term newborns, and we found participation coefficient contributed most to the separation of the two groups (Figure 5b).

## 4 DISCUSSION

The present study investigated the longitudinal developmental pattern of brain structural networks of preterm‐borns from birth to TEA, in terms of network architecture and nodal topology, and the impact of prematurity on structural networks when compared with term‐born neonates at birth. Our results revealed that (1) premature brain from birth to TEA experienced rapid development in network architecture, exhibited as reduced characteristic path length, clustering coefficient, and modularity, increased small‐worldness, and changes in hub organization (increases of connector hubs) and nodal properties; moreover, (2) preterm‐birth significantly altered the nodal properties based on clustering coefficient, within‐module degree, and participation coefficient measurements, rather than the global topological organization in terms of integration, segregation, and small‐worldness, at TEA when compared with the TB neonates; and these changes demonstrated high discriminative power in classifying the two cohorts. These results implied that although PB neonates showed comparable global network topology at TEA with TB neonates, the nodal properties remained largely abnormal, suggesting that premature exposure to the extrauterine environment could lead to atypical development of the structural network.

### 4.1 Increased integration and decreased segregation during the maturation of preterm brains to TEA

The increases in shortest path length and local clustering of the structural network coupled in the development of PB brains were consistent with previous studies showing similar alterations during both normal fetal (Song et al., 2017) and PB brain development (Ball et al., 2014; Liu et al., 2021; Sa de Almeida et al., 2021). Because clustering coefficient and the shortest path length reflected density of clustered connectivity around individual nodes and ability of communication between distributed brain regions, respectively (Rubinov & Sporns, 2010), these changes suggested that the PB brain tended to accelerate the information exchange across the network (global integration) and reduce barriers between locally segregated parcels with growth, resulting in an optimization of balance between segregation and integration (increased small‐worldness). This viewpoint was confirmed by a previous study indicating premature brain development was positively associated with global efficiency but negatively correlated with local efficiency (Batalle et al., 2017). A possible interpretation for this phenomenon would be the development of short‐ and long‐range fiber tracts during this period (increased fiber integrity), which facilitated the “handshake” of fibers to form a highly connected network (Métin & Godement, 1996; Molnár et al., 1998; Zheng et al., 2023). In addition, there was no significant difference between PB‐TEA and TB neonates in all of the global network properties, suggesting the development of premature brains from birth to TEA may prioritize the optimization of global topological architecture of the structural network. This phenomenon was also reported by another recent study based on data that are independent to the dHCP dataset (Jang et al., 2023).

Although our findings were supported by a series of previous studies showing decreased clustering measures and shortest path length during both normal fetal (Song et al., 2017) and preterm brain development (Ball et al., 2014; Liu et al., 2021; Sa de Almeida et al., 2021), there were also studies reported conflict results (e.g., increased local clustering) (Brown et al., 2014; van den Heuvel et al., 2015; Zhao et al., 2019). The inconsistency, we speculated, may be attributed to methodological confounds, such as sample size, acquisition protocols, analytic method (e.g., fiber tracking algorithm), definition of nodes and edges, etc., indicating the necessity of a validation study on a large sample set using a consistent analysis pipeline.

### 4.2 Preterm‐birth alters the nodal topology at TEA

Our results indicated that the PB brain exhibited more ‘immature’ regional topology at the time of full‐term birth, though the architecture of the whole brain almost reached the typical development level. Brain regions associated with high‐level cognitive functions (e.g., anterior cingulate cortex (ACC), insula, medial prefrontal cortex, precuneus, MTL, thalamus, and basal ganglia) showed significantly altered clustering (clustering coefficient), nodal importance (within‐module degree), or diversity (participation coefficient) in TB brains relative to PB‐TEA, whereas, no significant difference in clustering and diversity were found in perception (e.g., visual cortex) and somatosensory between the two groups. Since these brain regions were involved in multiple cognitive processes, such as attention, salience, and socio‐emotional processing (Uddin et al., 2017), modulation of emotional responses (assigning emotions to certain stimuli and making correct facial/vocal expression) (Bush et al., 2000), sensorimotor integration (Kreitzer & Malenka, 2008), learning and memory formation (Baars & Gage, 2013), and generation of consciousness (Alkire et al., 2008; Yu et al., 2021; Zheng et al., 2021), this observation was consistent with the maturation order of the brain after birth from primary to higher‐order cortices (Ouyang et al., 2017). Moreover, the increased inter‐module connectivity along with decreased clustering may suggest an integration process of neural information across distributed systems to support the emergence of diverse mental/cognitive states at later ages. On the other hand, these changes also provided network‐level evidence for the impaired cognition, emotion, and learning functions in the preterm cohort (Anderson & Doyle, 2003; Montagna & Nosarti, 2016; Spittle et al., 2009; Witt et al., 2014).

Interestingly, we showed high discriminative power of nodal properties in classifying PB‐TEA neonates from TBs, with over 96% accuracy and specificity of 100%, providing additional evidence for the abnormal regional topological organization in the PB‐TEA cohort that was a distinguishable feature of these neonates. Among the three types of nodal metrics, participation coefficient exhibited the highest discriminative power, suggesting premature birth may cause interruption or delayed development of the communication routes between communities at TEA. The brain regions with significantly affected participation coefficient (see Figure 2) mainly concentrated in orbitofrontal and temporal cortices, and the temporal–parietal junction of the left hemisphere, in line with previous findings indicating weakened connectivity strength in multiple intra‐hemispheric pathways linking limbic and paralimbic systems in PB‐TEA infants relative to TB neonates (Sa de Almeida et al., 2021).

### 4.3 Reassignment of hub functions optimized the communication routes both between communities

Our results confirmed the presence of modular organization of structural network in PB newborns, and the spatial distribution of communities maintained high consistency with the TB newborns, PB infants at TEA, and 1‐year‐old infants (Huang et al., 2015; Jang et al., 2023; Shi et al., 2011). Interestingly, the segregated orbitofrontal module in the PB‐AB cohort merged with the prefrontal community at TEA, which might suggest the immaturity of orbitofrontal neurodevelopment of PB newborns and rapid increases of connectivity between these two communities from birth to TEA. Previous studies have indicated that the third trimester is a crucial phase for the sulcogyral development of the orbitofrontal cortex (Ganella et al., 2015; Kringelbach & Rolls, 2004), and the formation of gyrification is likely driven by tension along axons (Van Essen, 1997). Because the orbitofrontal cortex is extensively involved in executive functioning, somatosensory stimuli decoding, and reward‐related behavior (Rolls, 2004), exposure to the ex utero environment during this critical period may increase the risk of abnormal development of the orbitofrontal cortex, which might be associated with the executive functioning deficits in children born preterm (Sandoval et al., 2021). Notably, it might be also possible that the isolation of the orbitofrontal module in the PB‐AB cohort was related to low image quality due to B0 inhomogeneity and susceptibility, although the data have been corrected for susceptibility‐induced bias and eddy‐current distortions using the dHCP preprocessing pipeline.

The identified network hubs were in line with previous literature (Sa de Almeida et al., 2021; van den Heuvel et al., 2015), comprising brain regions of limbic/paralimbic (insula, parahippocampus, and middle cingulate cortex) and sensorimotor (PreCG and PostCG) systems, and fusiform gyrus. The bilateral STG, identified as key provincial hubs in PB‐AB newborns, presented a low occurrence rate in both PB‐TEA and TB groups. This developmental trend was supported by (Sa de Almeida et al., 2021) showing disappeared temporal hubs (i.e., bilateral STG and left Heschl gyrus) in TB newborns relative to very preterm infants at TEA. Interestingly, a stable community structure was accompanied by a progressive reassignment of hub functions, manifested as a significant increase of connector hubs from preterm birth to TEA. Provincial hubs are important for within‐network communication, whereas connector hubs facilitate information flow between distinct brain networks (van den Heuvel & Sporns, 2013; Zheng et al., 2020). The increased connectors suggested an optimization process of the brain to build a more robust and efficient architecture, for example, bridging multiple segregated communities to facilitate inter‐community interactions and network integration (van den Heuvel & Sporns, 2013), which was in good accordance with the reduced characteristic path length.

### 4.4 Limitations

There were several limitations in the current study. First, the sample size of this study was limited. In this study, we have controlled for major factors that might influence the validity and reproducibility of our findings, including (1) strictly controlled data quality (considering image artifacts, radiology score, and QC reports provided by dHCP); (2) a well‐matched control group (each PB‐TEA infant had a TB newborn who was gender, age, and head circumference matched); and (3) use of standard graph theory analysis and network measures (Ratnarajah et al., 2013; Rubinov & Sporns, 2010). However, validation on larger independent datasets is still necessary to further examine the validity, reproducibility, and generalizability of our findings. Second, the community detection algorithm we used largely relies on the chosen resolution parameter that determines the scale of detected communities (He et al., 2018), though we performed a robust community segmentation pipeline that has been widely used in relevant studies (Cohen & D'Esposito, 2016; Lancichinetti & Fortunato, 2012; Zheng et al., 2019; Zheng et al., 2021), and the detected communities were in good accordance with previous literature (Shi et al., 2011). Whether the community parcellations were sensitive to the usage of different algorithms and the resolution parameters needed to be further explored, which is beyond the scope of this study. In addition, the dMRI data of dHCP is not isotropic. In the present study, images were resampled to 1.5 mm isotropic resolution to ensure a better representation of fiber orientation within voxels and the accuracy of fiber tracking (Basser et al., 2000); however, the interpolation during this process might lead to boundary blurring and loss of local details (Mishra et al., 2006; Yap & Shen, 2012), which is a common problem of 2D multislice dMRI and may affect the tractography results.

## 5 CONCLUSION

In conclusion, our results demonstrated that the development of preterm‐born neonatal brains from birth to TEA experiences evident reorganization of structural networks, for example, increased integration and decreased segregation. Moreover, preterm‐born significantly altered cerebral development in terms of nodal properties (i.e., nodal properties based on clustering coefficient, within‐module degree, and participation coefficient), but not global network architecture, compared with term‐born neonates at TEA. These results suggested a speeded development of the global organization of premature structural networks after birth, but preterm‐birth may still have a large impact on local network topology even at TEA.

## AUTHOR CONTRIBUTIONS

Weihao Zheng processed imaging data. Weihao Zheng and Xiaomin Wang performed all analyses with the preprocessed data. Weihao Zheng, Tingting Liu, and Dan Wu drafted the manuscript. All authors contributed to the interpretation and reviewing of the manuscript.

## FUNDING INFORMATION

This work was supported by the SIT2030‐Major Projects (2021ZD0202000, 2021ZD0200800, 2021ZD0200202), the National Key Research and Development Program of China (2019YFA0706200, 2018YFE0114600), and the National Natural Science Foundation of China (62227807, 62202212, 61801424, 81971606).

## CONFLICT OF INTEREST STATEMENT

The authors declare no conflict of interest.

## Supporting information


**FIGURE S1.** Comparison of binary network topology between groups. (a) Between‐group differences in clustering coefficient, the shortest path length, and small‐worldness. Global network metrics were computed based on binary networks with varying sparsities (retaining the strongest 10%–25% of links in 5% increments). (b) The normalized clustering coefficient (Gamma) and normalized shortest path length (Lambda) in the three groups. Gamma and Lambda were calculated as the ratio to values of a randomly rewired null model. Networks in all the three groups showed similar Lambda and higher Gamma than a randomly rewired network, suggesting a small‐world architecture of these networks. *FDR *q* < 0.05 by paired *t*‐test for longitudinal analysis and ANCOVA for PB‐TEA vs. TB.
**FIGURE S2.** Effects of head motion on network statistics. (a) Absolut head motion of the three groups. (b) Comparison of global network metrics after controlling for motion artifacts. *p < .05, **p < .01, ***p < .001.
**TABLE S1.** Summary of the image quality metrics calculated by the EDDY QC tools.

## Data Availability

We thank the numerous contributors to the dHCP database for their effort in the collection, organization, and sharing of their datasets. The data that support the findings of this study are openly available in the dHCP at http://www.developingconnectome.org/second-data-release/.
