# Supplementary material for: Preterm‐birth alters the development of nodal clustering and neural connection pattern in brain structural network at term‐equivalent age
Source: Hum Brain Mapp. 2023 Aug 4;44(16):5372–86. doi: 10.1002/hbm.26442 (PMC10543115; doi:10.1002/hbm.26442)
Supplement: Supplementary file 1 — FIGURE S1. Comparison of binary network topology between groups. (a) Between‐group differences in clustering coefficient, the shortest path length, and small‐worldness. Global network metrics were computed based on binary networks with varying sparsities (retaining the strongest 10%–25% of links in 5% increments). (b) The normalized clustering coefficient (Gamma) and normalized shortest path length (Lambda) in the three groups. Gamma and Lambda were calculated as the ratio to values of a randomly rewired null model. Networks in all the three groups showed similar Lambda and higher Gamma than a randomly rewired network, suggesting a small‐world architecture of these networks. *FDR q < 0.05 by paired t‐test for longitudinal analysis and ANCOVA for PB‐TEA vs. TB. FIGURE S2. Effects of head motion on network statistics. (a) Absolut head motion of the three groups. (b) Comparison of global network metrics after controlling for motion artifacts. *p < .05, **p < .01, ***p < .001. TABLE S1. Summary of the image quality metrics calculated by the EDDY QC tools. [file HBM-44-5372-s001.docx]

**Supplementary**


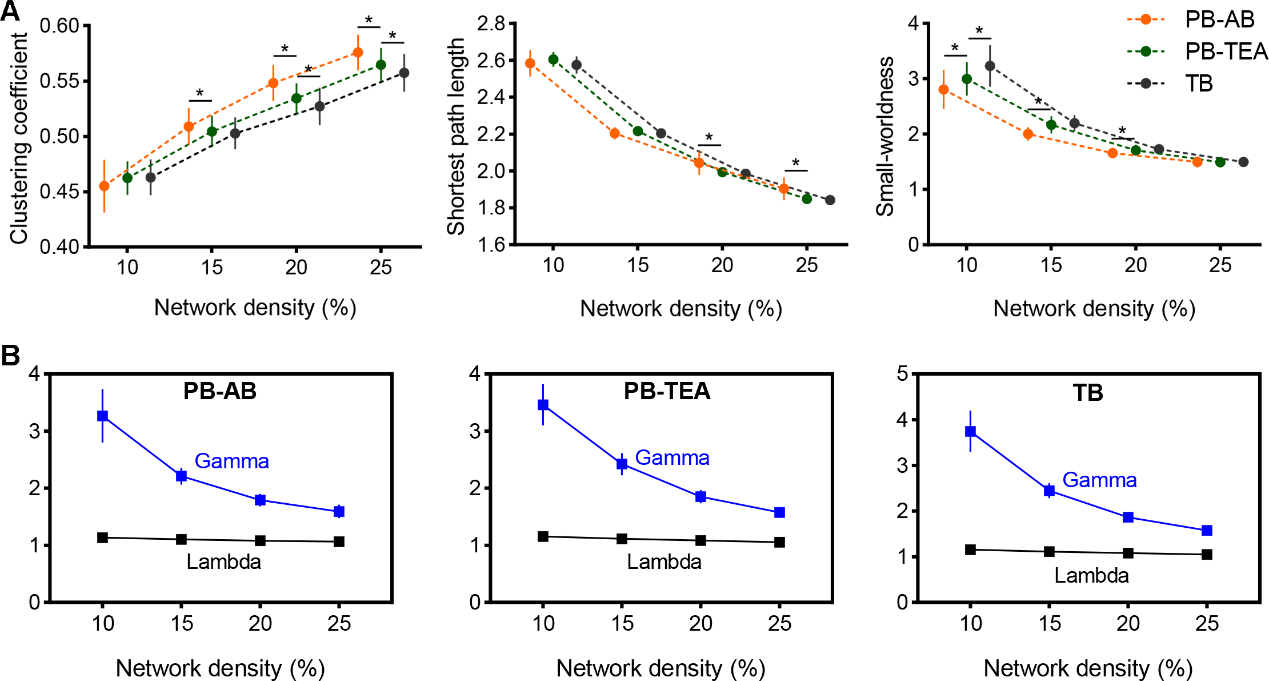


**Figure S1**. Comparison of binary network topology between groups. (**A**) Between-group differences in clustering coefficient, the shortest path length, and small-worldness. Global network metrics were computed based on binary networks with varying sparsities (retaining the strongest 10–25% of links in 5% increments). (**B**) The normalized clustering coefficient (Gamma) and normalized shortest path length (Lambda) in the three groups. Gamma and Lambda were calculated as the ratio to values of a randomly rewired null model. Networks in all the three groups showed similar Lambda and higher Gamma than a randomly rewired network, suggesting a small-world architecture of these networks. * FDR *q* < 0.05 by paired t-test for longitudinal analysis and ANCOVA for PB-TEA vs. TB.


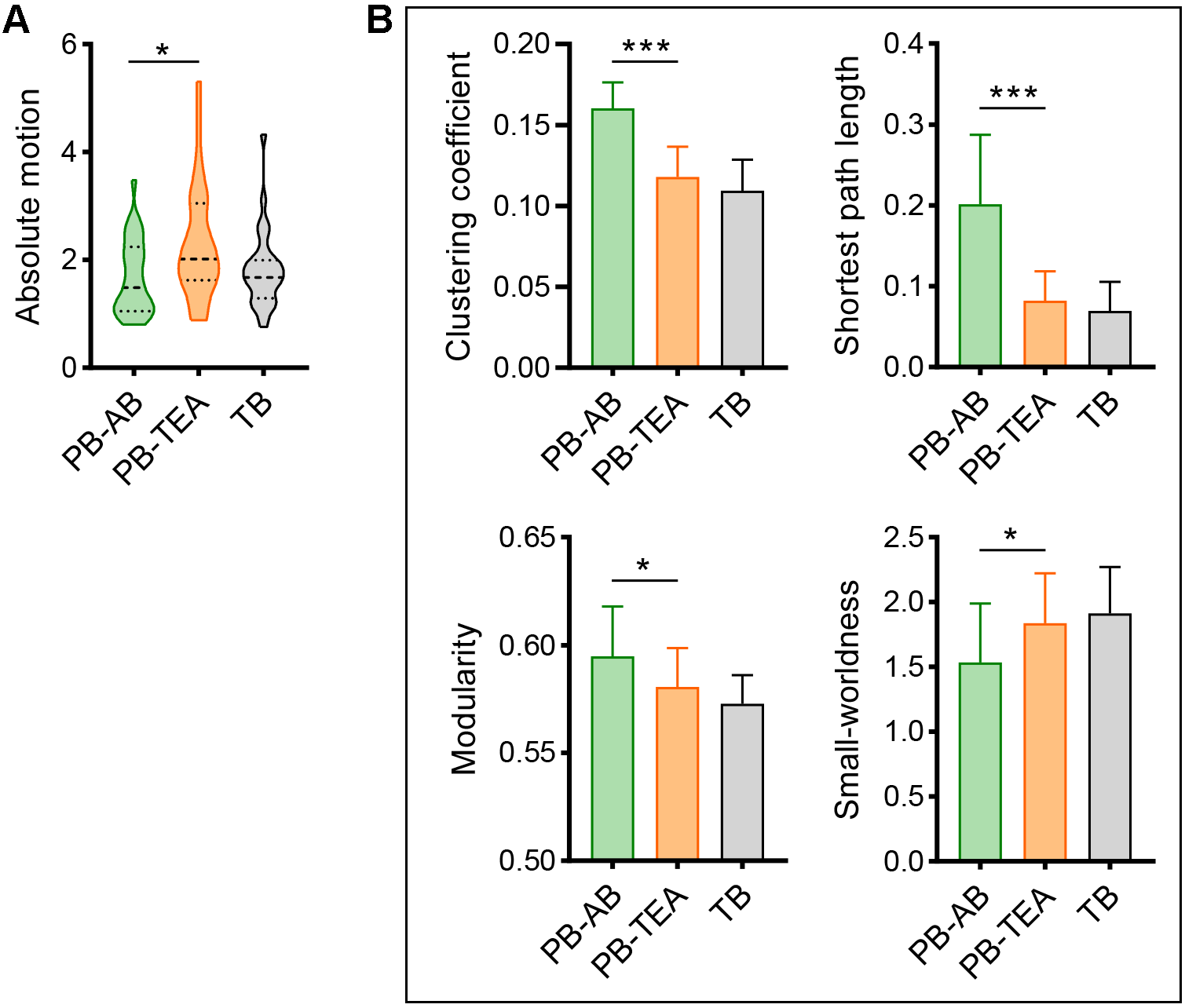


**Figure S2**. Effects of head motion on network statistics. (**A**) Absolut head motion of the three groups. (**B**) Comparison of global network metrics after controlling for motion artifacts. * *p* < 0.05, ** *p* < 0.01, *** *p* < 0.001.

**Table S1**. Summary of the image quality metrics calculated by the EDDY QC tools

| Group | Absolute motion (mm) | Relative motion (mm) | Outlier slices (%) |
| --- | --- | --- | --- |
| PB-AB | 1.65 ± 0.64 | 1.54 ± 0.65 | 3.88 ± 1.22 |
| PB-TEA | 2.26 ± 1.04 | 2.15 ± 1.23 | 5.01 ± 1.91 |
| TB | 1.83 ± 0.73 | 1.71 ± 0.63 | 4.83 ± 2.07 |
